# Teaching Essential EMG Theory to Kinesiologists and Physical Therapists Using Analogies Visual Descriptions, and Qualitative Analysis of Biophysical Concepts

**DOI:** 10.3390/s22176555

**Published:** 2022-08-30

**Authors:** David A. Gabriel

**Affiliations:** Electromyographic Kinesiology Laboratory, Faculty of Applied Health Sciences, Brock University, 1812 Sir Isaac Brock Way, St. Catharines, ON L2S 3A1, Canada; dgabriel@brocku.ca; Tel.: +1-(905)-688-5550 (ext. 4667); Fax: +1-(905)-688-8364

**Keywords:** muscle fiber, motor unit, compound muscle action potential, monopolar and bipolar surface electrode configuration, volume conduction

## Abstract

Electromyography (EMG) is a multidisciplinary field that brings together allied health (kinesiology and physical therapy) and the engineering sciences (biomedical and electrical). Since the physical sciences are used in the measurement of a biological process, the presentation of the theoretical foundations of EMG is most conveniently conducted using math and physics. However, given the multidisciplinary nature of EMG, a course will most likely include students from diverse backgrounds, with varying levels of math and physics. This is a pedagogical paper that outlines an approach for teaching foundational concepts in EMG to kinesiologists and physical therapists that uses a combination of analogies, visual descriptions, and qualitative analysis of biophysical concepts to develop an intuitive understanding for those who are new to surface EMG. The approach focuses on muscle fiber action potentials (MFAPs), motor unit action potentials (MUAPs), and compound muscle action potentials (CMAPs) because changes in these waveforms are much easier to identify and describe in comparison to the surface EMG interference pattern (IP).

## 1. Introduction

Instructors in surface electromyography (EMG) often have expertise in the area as researchers who use surface EMG techniques or those who conduct research aimed at advancing surface EMG signal processing methods and/or biomedical instrumentation. It can be a challenge to prepare lesson plans for students with diverse backgrounds when the effective use of surface EMG methodology and its interpretation requires a common frame of reference [1]. For example, kinesiologists in an EMG course may be planning for advanced study in physiology, biomechanics, or motor behavior. Likewise, physical therapists may have an undergraduate degree from a variety of disciplines, ranging from biology to physics, as long as the prerequisites for admissions are met.

Both kinesiologists and physical therapists may have taken undergraduate courses in calculus and physics, but other courses in their general curriculum will not have required an intensive application of that knowledge. This paper outlines a pedagogical approach for teaching foundational concepts in surface EMG to students in kinesiology and physical therapy that uses a combination of analogies, visual descriptions, and qualitative analysis of biophysical concepts to develop an intuitive understanding. The goal is to form a bridge to the material appearing in surface EMG texts [2,3,4] and reference books [5,6], and recent work by the International Society of Electrophysiology and Kinesiology (ISEK) in providing guidelines [7], tutorials [8,9], and consensus papers [10,11,12,13] that are ideal for those who are new to surface EMG. Thus, no new scientific knowledge or new technology in the surface EMG field will be presented.

It is assumed that students in the allied health sciences have a strong background in physiology. The approach focuses on muscle fiber action potentials (MFAPs), motor unit action potentials (MUAPs), and compound muscle action potentials (CMAPs) because changes in these waveforms are much easier to identify and describe in comparison to the surface EMG interference pattern (IP), with the understanding that the same principles extend to the surface EMG IP. Equally important, there is a large gap between clinical and kinesiological electromyographers concerning electrode placement, sign conventions, and nomenclature that will be addressed, because evoked potentials are an important part of a kinesiological investigation of the neuromuscular system. Monopolar recordings will serve as the building block for motivating the theory of bipolar surface EMG detection to provide a common frame of reference. The following sections constitute a teaching progression that starts with the basic principles of differential amplification and ends with a review of the fundamental theories for recording the bipolar surface EMG signal.

## 2. Differential Amplification

**Figure 1:**
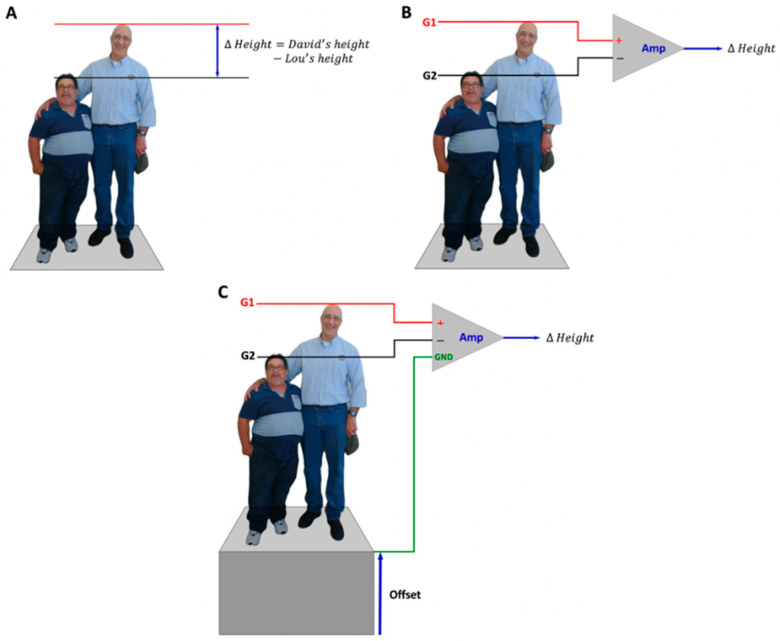
The differential amplification of height. (**A**) David’s height is indicated by the red (+) line and Lou’s height is indicated by the black line (−). (**B**) The height indicators are now depicted as electrode leads with G1 and G2 inputs to the operational amplifier, which sums the two heights as described in panel (**A**). The resulting output is the height difference. (**C**) The ground is now introduced to account for an “offset” where the height comparison is made above ground.

When comparing the relative magnitudes of two quantities, people intuitively perform a simple mathematical operation that involves subtracting one quantity from the other to evaluate the difference between them. Consider the picture in Figure 1A that shows the author with his friend Lou. The first thing that is noticed is the difference in height between the two individuals. The electric circuit that is used to compare two quantities and measure the difference between them is called an operational amplifier (op. amp.). The operational amplifier is symbolized in electronic schematics as a side-ways triangle, as depicted in Figure 1B.

The height analogy is now extended so that the two individuals represent the voltage applied to, or current entering into, the leads entering the terminals of an operational amplifier. One lead enters the G1 (red) terminal, which has a positive sign (+) because the signal is unaltered by the circuit. The second lead enters the G2 (black) terminal where the signal is inverted, which is the same thing as multiplying the values by a negative one. The G2 terminal is therefore given a negative sign (−). Thus, the operational amplifier compares the two signals by inverting one of the signals to determine the difference between them. The labels G1 (or, E1) and G2 (or, E2) are legacy identifications from a time when each electrode required a separate electronic grid of vacuum tubes (hence, the “G”), but it is still a common and convenient way to describe electrode placement procedures [14].

In this example, the individuals in Figure 1A,B are standing on the ground, so the height comparison occurs against the background of an absolute zero. Consider the situation where both individuals are standing on a platform (Figure 1C). The brain automatically estimates how far the platform is off the ground, and then subtracts out the “non-zero” offset from the height comparison. The third input terminal of the operational amplifier circuit is called the ground (GND) and it measures the non-zero offset against which signals entering G1 and G2 are compared. The non-zero offset is due, for example, to “common” electromagnetic fields that induce the flow of current over the surface of the skin. 

Differences in height between individuals may only be a few millimeters, barely perceptible to the eye, while the “offset” height may be many centimeters. In electrophysiology, the difference in electric potential between two points may be on a scale of a few microvolts or millivolts. A second function of the operational amplifier is therefore to increase the gain of the difference in electric potential between two points so that the signal can be measured and quantified on a computer. The signal can be multiplied with a gain from 10 to 10,000 times, analogous to the use of different magnification lenses on a microscope, to view the cellular material based on the size of the object being viewed.

### Comparing the Electric Potential at Two Different Points

**Figure 2:**
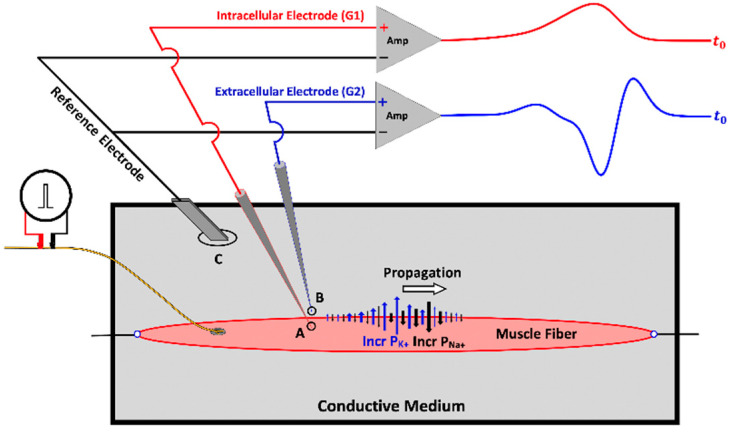
Differential amplification of the intracellular (A) and extracellular recordings (B). The reference electrode (C) in this figure is also the ground; it records the magnitude of the offset potential of the conductive medium that is used to compare the intracellular and extracellular potentials. The blue arrows indicate increased permeability for potassium ions (P_K+_) and black arrows indicate increased permeability for sodium ions (P_Na+_). G1 records the intracellular potential while G2 records the extracellular potential. The positive sign for both G1 and G2 denotes that the inputs are unaltered. The reference electrode is connected to the inverting inputs of the amplifiers because it represents the offset that is subtracted from the G1 and G2 inputs.

Figure 2 serves as a bridge for understanding the different electrode configurations for intracellular and extracellular recordings with the common reference electrode introduced in Figure 1. The example shows the intracellular (G1) and extracellular (G2) electrodes as separate recordings that are individually compared to a common reference to assess their relative magnitudes. Later, a connection is made with bipolar recordings where G1 and G2 are placed on the muscle and the reference electrode is the ground electrode (GND).

A review of extracellular recordings is a convenient starting point because the electrochemical events measured from within the cell result in a reciprocal change in charge outside the cell (i.e., an influx of Na^+^ into the cell results in a positive potential at G1, but a negative potential at G2 in the surrounding extracellular fluid). Recording these electrochemical events using a monopolar electrode configuration is a good starting point for three reasons: (1) it is easy to connect changes in extracellular potentials to what is recorded from an electrode placed on the skin surface, which (2) can be extended to monopolar recordings of the CMAP that are recorded over the motor point, because (3) the CMAP is integral to the clinical surface EMG and an important methodological control in kinesiological experiments.

## 3. Muscle Fiber Action Potential

The time-evolution of the intracellular action potential is so ingrained in the undergraduate curriculum that it is necessary to reinforce the concept of its spatial extent on the muscle membrane. The following example adapted from Loeb and Gans [15] can be used to solidify the simultaneous spatial and temporal representations of the MFAP. Assume that depolarization starts at a single point on the muscle membrane. The current then begins to flow across the membrane and along it in loops, depolarizing nearby regions and widening the depolarized region at the speed of 4 m/s (or 4 mm/ms). If the total duration of the depolarization phase is 0.5 ms, then the spatial extent (distance) on the muscle membrane that it occupies can be calculated by knowing the distance (d) formula. Since distance equals velocity (v) multiplied by time (t), the spatial extent of the depolarization phase is 2 mm. For the sake of simplicity, the repolarization phase is assumed to have the same duration as depolarization. The complete action potential, therefore, occupies a 4 mm patch on the muscle membrane, for a total of 1 ms. The negative and positive charges are then assumed to be situated in the middle of their respective regions. Since the depolarization and repolarization phases are immediately adjacent to each other, the distance between charges (dipole distance) is assumed to be 2 mm. The symmetric dipole is a simple but incomplete biophysical representation of the MFAP that is convenient for illustrating EMG theory. A more realistic depiction of the MFAP dipole involves a repolarization phase that is longer than the depolarization phase [16,17]. As will be described later, the asymmetric shape is better represented by a second current dipole that combines with the first to form a tripole [18].

### 3.1. The Propagating Component of the MFAP

**Figure 3:**
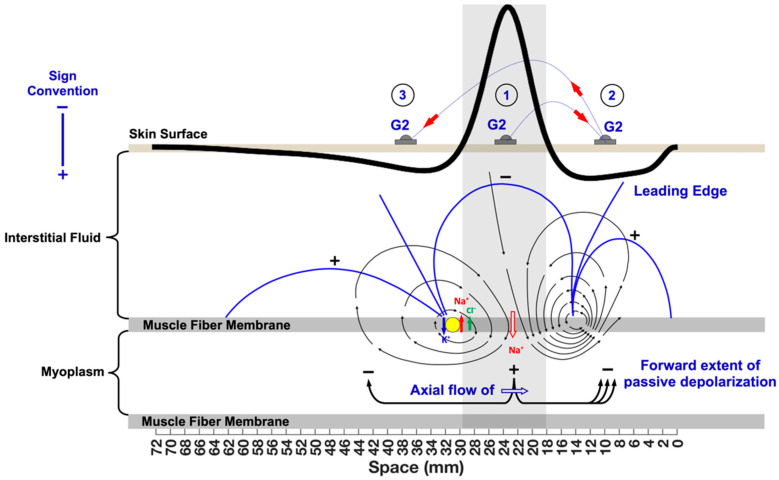
The propagating components of the muscle fiber action potential (MFAP). The MFAP is depicted with its corresponding current lines (black lines going from the + pole to the − pole) and equipotential lines (blue lines surrounding each pole) originating from the dipole. The locations of G2 on the skin surface are: 1—over the depolarization zone; 2—the leading edge; and 3—the repolarization zone. The muscle fiber–tendon end-effect is circled and is represented by equipotential lines compressing at the muscle–tendon junction immediately above. This is an approximation since the actual action potential is better modeled as a double dipole (a tripole).

The propagation velocity of the MFAP depends on the dynamics of the opening and closing of the Na^+^, K^+^, and Cl^−^ channels (which are possibly changed by pathologies). The much greater velocity for charges flowing through the extracellular medium results in the depolarization and the repolarization dipoles having a much larger appearance (Figure 3). The dipoles depicted on the muscle membrane are only a representation of the current sources. For this example, imagine that Figure 3 is a “snapshot” of the MFAP at a single moment in time as it occupies a certain segment of length on the muscle fiber, and we can move G2 to measure the extracellular potential at specific points over the muscle fiber to explore the charge distribution over its membrane. Since G2 is the “inverting” electrode, the depolarization phase is displayed above the horizontal zero line (G1 and the reference electrodes are not shown). Clinical electromyographers are trained to view evoked potentials with the negative polarity above the horizontal because it facilitates the identification of the onset of waveforms [14,19]. Figure 3 follows the convention in clinical EMG to start forming a bridge between conventions adopted in electrophysiology and engineering while introducing students to a spatial understanding of the MFAP.

It is assumed that the neuromuscular junction (NMJ) is in the middle of the muscle fiber (x = 0 mm) and that the locations of G2 are between the NMJ and the tendon (x = 65 mm). If the G2 electrode is placed directly over the site of depolarization (location #1), it will record a negative potential as Na^+^ ions leave the immediate region and rush inside the cell. At the same time, Na^+^ ions within the interstitial fluid in front of the depolarization zone are attracted to this central region. If the G2 electrode is placed in front of the depolarization zone (location #2), the passive flow of Na^+^ will be recorded as a weak positivity, termed “the leading edge”. Note that the direction of the arrows of the current lines is pointed toward the depolarization zone. As might be expected, if the G2 electrode is placed behind the depolarization zone (location #3), a strong positivity is recorded because Na^+^ ions are actively pumped back into the interstitial fluid over this segment of the muscle fiber. Taken together, the sign of the potentials detected by G2 at the three different locations shows that the MFAP can be represented by a current tripole.

#### Muscle Fiber Conduction Velocity

The velocity of MFAP propagation along the muscle membrane is determined by the speed at which the Na^+^ and K^+^ channels open and then later close [20]. Like domino blocks, the speed of the wave depends on how fast a block detaches from the floor and hits the next piece. In the absence of changes in the peripheral conditions of the muscle (i.e., temperature or fatigue), the channel kinetics remain constant. However, muscle fiber conduction velocity (MFCV) also scales with its diameter [21].

**Figure 4:**
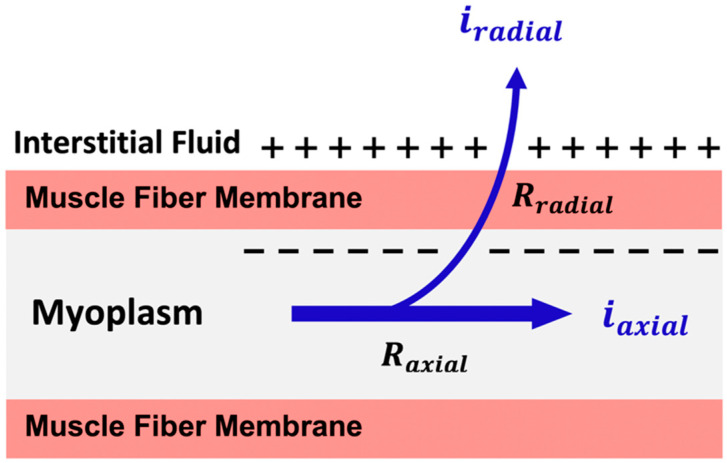
An illustration of the orientations of the axial flow of current (i_axial_) and its resistance (R_axial_) relative to the outgoing current radially through the muscle membrane (i_radial_) and its resistance (R_radial_) as both contribute to the capacitive properties of the muscle fiber.

The advance of the leading edge is determined by two factors, as illustrated in Figure 4. First, is the resistance (R_axial_) to the axial flow of current inside the muscle fiber (i_axial_), which is a function of the resistivity of both the myoplasm and muscle fiber diameter [22]. Think of how hard it is to drink a milkshake through a straw that is meant for a soft drink. A larger diameter straw is used because it has a decreased axial resistance to the flow of a more viscous fluid. The second factor is the flow of current through the muscle membrane (i_radial_). Radial resistance (R_radial_) to the flow of current through the membrane depends on the surface area, which is another size-dependent factor. Both the axial and radial properties determine the capacitive properties of a muscle fiber, which is the ability to hold a charge [23]. Once initiated, the action potential starts to decay in unmyelinated nerve and muscle fibers, and the capacitive properties determine the distance (mm) it can travel and still regenerate. The diameter has a significant impact because it allows the axial current to flow farther before the outgoing current is insufficient to maintain the membrane above the threshold and trigger depolarization.

The axial flow of current is labelled as the “forward extent of passive depolarization” in Figure 3. The blue lines illustrate that Na^+^ ions rushing inside the muscle fiber are attracted forward toward the negativity inside the cell. At the same time, the current lines on the outside of the muscle membrane show that the leading edge is associated with the attraction of positive ions within the interstitial fluid toward the depolarization zone. As a result, the voltage across the membrane associated with the leading edge is closer to the threshold and the action potential regenerates more easily, which increases the conduction velocity in larger fiber diameters even though channel kinetics remain unchanged [24].

The surface EMG can be used to measure the distribution of the MFCVs of motor units (MUs) that are active during voluntary contractions [25,26,27,28,29]. The basis of the technique involves knowing the distance between two electrodes and the time that it takes action potentials to travel between them [30]. The normal range of MFCV is between 3 and 6 m/s [30,31]. Since channel kinetics are sensitive to temperature and metabolic by-products generated during fatigue [21,32], MFCV is also used to monitor peripheral changes within the muscle during different interventions [28,33,34,35] and to account for factors that would affect the interpretation of the surface EMG signal [36,37]. Non-invasive determination of MFCV is also advancing in the electrodiagnostic assessment of myopathic disorders related to channelopathies, inflammatory responses [38,39], and altered muscle function with the progression of diabetic neuropathy [40], which are typically studied with needle electrodes [41,42].

### 3.2. The Non-Propagating Component on the MFAP

**Figure 5:**
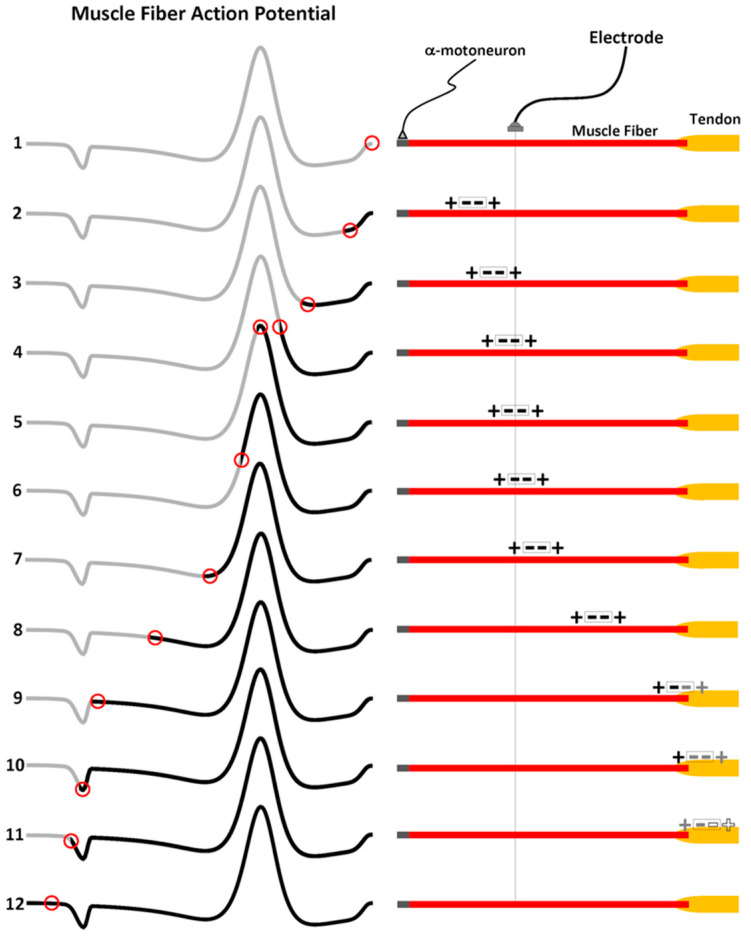
The development of the non-propagating component of the muscle fiber action potential (MFAP) is shown in the left panels, and the travelling double dipole (or, quadrupole) is depicted in the right panels. As the quadrupole propagates from left to right, there is an increase in the length of the thick black line over the grey trace of the MFAP to simulate its progression. The red circle on the MFAP shows the potential recorded at the electrode due to the travelling quadrupole. The convention for the polarity of the MFAP is positive below the horizontal and negative above the horizontal.

The MFAP also consists of a non-propagating component that arises from the end of fiber (EOF) effects. The EOF effect is better explained if the MFAP tripole described in Section 3.1 is represented as two current dipoles with the negative ingoing currents stacked at the center (+ = +), which is a quadrupole [43,44,45]. However, to facilitate a graphic depiction of the EOF effects, the depolarization phase of the MFAP is divided in two at the peak. The front half of the MFAP consists of one negative charge and the positive charge associated with passive depolarization termed the leading dipole (LD). The back half of the MFAP is called the trailing dipole (TD); it consists of the other negative charge and the strong current source associated with the repolarization. Thus, for visual purposes, the tripole has been “unstacked” to represent the MFAP as two adjacent dipoles (+ − − +) in Figure 5.

The right panels of Figure 5 show a single muscle fiber–tendon unit. A resting state is depicted in trace 1. Traces 2 through 11 then show the quadrupole as it propagates (left-to-right) from the NMJ in the middle of the muscle fiber to the muscle–tendon junction at the other end. The vertical line underneath the electrode provides a reference for what portion of the quadrupole (TD–LD) is dominating the potential recorded at the electrode as it changes position along the muscle fiber. The left traces of Figure 5 show the progression of the MFAP. The first MFAP trace is completely grey to indicate the current resting state (red circle) before the generation of the action potential (future states 2 to 8). The black lines represent the potential that is recorded at the electrode with each advance of the quadrupole along the muscle fiber up until that point. Thus, the increase in length of the black line simulates the evolution of the MFAP as it propagates from left to right. The red circle on the MFAP corresponds to the potential that is recorded at the electrode due to each position of the quadrupole along the muscle fiber. Since the red circle represents a fixed point in space (the electrode), an equivalent representation is obtained by aligning the red circles with the vertical line bisecting the muscle fiber to the right of its corresponding black trace in Figure 5.

As the LD enters the proximity of the electrode, the positive polarity of the leading edge is formed (traces 2 and 3). The negative charge of the LD starts to form the front half of the depolarization phase (trace 4). The peak of the depolarization phase is achieved when the negative charges of the LD and TD are centered beneath the electrode (trace 5). When the electrode detects the beginning of the TD, the negative potential decreases because the positive charge is nearby (trace 6). The repolarization peak is achieved when the positive charge is directly below the electrode (trace 7). As the MFAP continues, the TD has the greatest impact on the potential as it propagates farther away from the electrode. The potential recorded at the electrode is progressively less positive as both charges of the TD start to contribute more equally at farther distances until the LD has reached the muscle–tendon junction (traces 8 and 9). When the negative charge of the TD has moved across the muscle–tendon junction, the positive charge now dominates the potential recorded at the electrode. The electric potential is recorded as a minor positivity toward the tail end of the MFAP because it is far away (trace 11). The completed MFAP is now depicted by an entirely black waveform (trace 12). The EOF effects are also evident in MUAPs and CMAPs but are not distinguishable in the single channel surface EMG IP, but they are nonetheless present and contribute to the high-frequency content of the surface EMG signal [46].

## 4. Motor Unit and Compound Muscle Action Potentials

**Figure 6:**
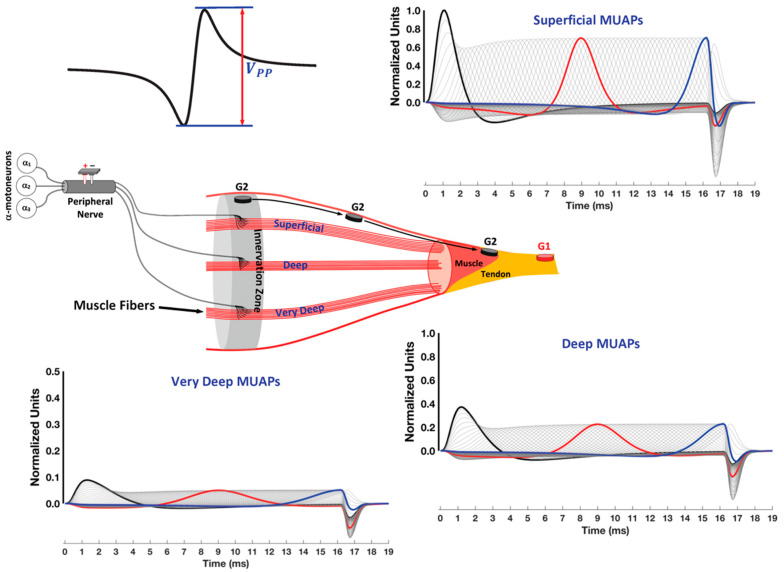
Monopolar recordings of changes in the motor unit action potential (MUAP) shape as the location of G2 is moved longitudinally from the motor point toward the muscle–tendon junction. The black, red, and blue lines correspond to G2 locations at the innervation zone (IZ) mid-point and close to the muscle–tendon junction. The grey lines show the shapes of the MUAPs for incremental G2 locations in between the black, red, and blue lines. Rotating clockwise, starting from the top of the figure, the MUAPs associated with superficial, deep, and very deep muscle fiber locations, respectively, are depicted. The amplitude of the MUAPs in all three graphs is normalized with respect to the peak value of the depolarization phase for the most superficial muscle fibers underneath G2 on the IZ. The G1 electrode is placed on the tendon and serves as the reference electrode for G2. The ground electrode that measures the “offset” potential is not shown.

For this example, imagine that the CMAP has been evoked by electrical stimulation of the peripheral nerve and that superficial, deep, and very deep MUs can be selectively recorded (Figure 6). The terms used for MU depth are arbitrary distances to illustrate changes in the MUAP shape with progressively deeper locations within the muscle. To the left of the muscle, is the peripheral nerve. The stimulation probe above the peripheral nerve is oriented with the cathode toward the muscle for orthodromic motor nerve conduction [47]. All MUs within the stimulated muscle undergo synchronous depolarization and repolarization, summating to generate the CMAP. The CMAP is also referred to as the motor wave (M-wave). In each subplot of the figure, the black, red, and blue MUAPs depict what is recorded at the IZ, halfway between the IZ and tendon, and close to the muscle–tendon border, respectively. The grey lines show the incremental changes in electrode position in between the colored MUAP traces.

### 4.1. Innervation Zone versus Motor Point

The innervation zone (IZ) and motor point (MP) have anatomical and operational definitions [48]. The anatomical definitions are based on the muscle biopsies, electrical stimulation by a needle electrode, and cadaveric dissections to identify the most efficient locations for chemical denervation of the muscle, functional electrical stimulation, and a viable reinnervation zone for muscle transfers [48,49,50,51,52]. The IZ is a well-defined narrow band on the muscle where the motor neurons branch out to contact motor endplates (or, neuromuscular junctions) of the muscle fibers for each motor unit. The location of the IZ is more centrally within the muscle to allow the spread of action potentials and is identified using electrode arrays as the origin of MUAPs propagating bidirectionally toward the distal ends of the muscle [53,54]. Anatomically, the MP has been described as the entry point where the motor nerves enter the muscle before branching out within the muscle to contact neuromuscular junctions (NMJs). However, the anatomical definition of the MP is at variance with the operational definition, which is a small area over the skin surface that is the most sensitive to electrical stimulation [55,56]. Electrical stimulation of the MP will result in a barely visible muscle twitch with the lowest possible level of current [30,57,58]. Thus, it makes sense that the MP may be anatomically associated with the location of a very superficial NMJ [48].

Recent work has carefully demonstrated that the IZ and MP are not to be confused as having overlapping locations [59]. It has also been shown that there is variability between individuals in both the number and location of both IZs and MPs [59,60,61,62]. Clinical electromyographers are trained to know the general locations of motor points for muscles, which have been reported in textbooks [63,64,65]. With advances in electrode arrays to document the origin of MUAPs, a textbook now exists to help identify IZs for the correct electrode placement [3]. During electrodiagnostic testing, the clinician can quickly evaluate the electrode location based on the amplitude and shape of the CMAP on the screen of a diagnostic machine, then relocate the electrode if necessary [66]. However, kinesiological electromyographers must verify the location of the IZ “before” the application of surface electrodes [67,68].

### 4.2. Factors That Affect Waveform Shape

#### 4.2.1. Monopolar Recordings and Electrode Placement

A monopolar electrode configuration is typically preferred for electrodiagnostic testing [14] and is used here only as a building block for understanding the bipolar electrode configuration used in kinesiological electromyography. One electrode (G2) is placed on the IZ and the other (G1) is placed on the tendon associated with the muscle (see Figure 6). The G2 electrode is used to measure changes in the extracellular potential. The G1 electrode is placed on the tendon (non-excitable tissue) where the EMG of the muscle (including its EOF effect) is considered to be near zero. Thus, G1 represents a baseline against which changes in the potential at G2 are compared and the difference is amplified [66,69]. This electrode configuration is used to determine the nerve conduction velocity (NCV).

Electromagnetic fields induce the flow of current along the skin. The result is an “offset” potential recorded by the ground electrode (not shown). The magnitudes of both G1 and G2 are determined against this “offset” before obtaining the difference between them. Figure 6 shows that if G2 is placed on the IZ, the electrode will first detect a large depolarization phase across all fiber depths. All waveforms are normalized to the peak amplitude of the depolarization wave of the most superficial MU recorded at the IZ because the peak-to-peak amplitude (*V_PP_*) is greatest for this initial condition (see Figure 6). As G2 is moved away from the IZ toward the tendon, the *V_PP_* decreases in amplitude.

#### 4.2.2. Propagating and Non-Propagating Components and Crosstalk

As might be expected, the *V_PP_* of both the propagating and non-propagating components of the MUAPs exhibit a progressive decrease with an increase in electrode-source distance (Figure 6). The phrase “electrode-source distance” is used to describe the distance between the electrode and the “source” of the action potentials within the muscle [70]. A more subtle change is that the relative amplitudes of the two MUAP components reverse. That is, as the muscle fiber depth increases, the *V_PP_* of the non-propagating component becomes larger as a percentage of the *V_PP_* of the propagating component [71].

Electromyographic kinesiology has long focused on propagating potentials as the origin of crosstalk from neighboring muscles [72,73,74,75]. Placing the bipolar electrodes more centrally on the muscle away from its borders has been recommended to minimize contamination from this form of crosstalk [76]. More recent work has identified non-propagating potentials as a unique source of high-frequency crosstalk [46]. The reason is that the non-propagating potentials increase in amplitude relative to the propagating components as the electrode-source distance increases. High-frequency crosstalk associated with non-propagating potentials cannot be eliminated with a bipolar electrode configuration [77]. Figure 6 illustrates that regardless of where the electrode is placed over the muscle, the muscle-tendon end-effects have the same shape and the same spatial location, at the same point in time [71,77], because they are “far-field” potentials that are conducted through the limb volume [43,45], hence the term non-propagating. The *V_PP_* of non-propagating potentials only increases or decreases based on the electrode location and MU depth within the muscle [71,77]. The next section will illustrate that bipolar recordings can be represented as two-time delayed monopolar signals, one recorded at G1 and the other at G2, as long as the source is propagating. However, the two signals are synchronous concerning the occurrence of the end of fiber effect. As a result, the *V_PP_* of non-propagating potentials can be reduced by the single differentiation associated with a bipolar electrode configuration, which is also referred to as a single differential (SD) recording.

## 5. Bipolar Recordings Represented as Two Time-Delayed Monopolar Signals

**Figure 7:**
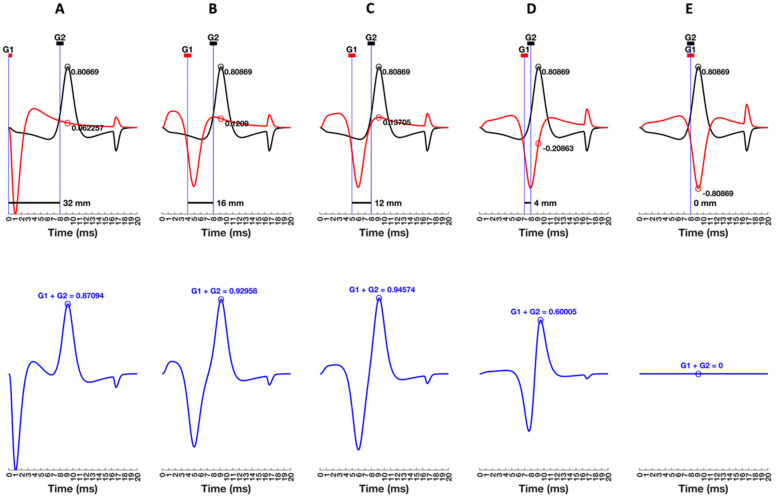
The monopolar recordings of a motor unit action potential (MUAP) by two different electrodes, G1 and G2. The location of G2 remains between the innervation zone (IZ) and the muscle–tendon junction, while G1 is moved closer to G2 to decrease the distance between them (top panels (**A**–**E**)). The bottom panels show the difference between the two monopolar MUAP recordings to construct a bipolar MUAP, as would occur with differential amplification, for each distance between G1 and G2. The sign conventions are G1 (+) and G2 (−), which means that the input to G1 is unaltered and the input to G2 is inverted by the op-amp and now displayed so the depolarization phase is positive above the horizontal zero line. The *x*-axis is the time-evolution of the MUAP in milliseconds for CV = 4 m/s.

Figure 7 depicts the same monopolar MUAP recorded at G1 and G2 while the inter-electrode distance (IED) decreased (panels A–E). The G2 electrode is held constant, midway between the IZ and the muscle–tendon junction so the shape of the MUAP recorded at G2 remains constant across panels. Notice that the shape of the MUAP recorded at G2 is the same as that for the middle electrode location (red line) in Figure 6. Similarly, the MUAP recorded by G1 placed at the IZ in Figure 7A has the same shape as the MUAP recorded at the first electrode location (black line) in Figure 6. The bipolar MUAP is then given as the simple summation of the two monopolar signals, which is presented in the panel immediately below. The process is analogous to using an Excel spreadsheet. The two separate monopolar data streams would be in the first two rows. The third row would be the bipolar MUAP obtained by the simple addition of G1 plus (−G2) given in the first two rows.

### 5.1. Understanding the G1 and G2 Sign Conventions

To avoid confusion, it is important to match the polarity of the potentials with inputs to the amplifier. Figure 7 follows the standard convention where G1 is (red +) and G2 is (black −). The negative depolarization phase propagating under G2 will be displayed above the horizontal zero line because the negativity is inverted (i.e., multiplied by −1), creating a positive deflection. When the depolarization phase travels under G1, it is not inverted and will still be displayed below the horizontal zero line. All waveforms are normalized to the peak amplitude of the depolarization wave of the most superficial MU recorded at the IZ because the peak-to-peak amplitude (*V_PP_*) is greatest for this initial condition. The peak amplitude of the MUAP recorded at the IZ is 1.0 units. The peak amplitude of the MUAP recorded at G2 midway between the IZ and the muscle–tendon junction is 0.80869 units (black circle). Panel A shows that when the peak MUAP amplitude at G2 is 0.80869 units, the value of the MUAP at G1 is 0.062257 units (red circle). The summation of the two normalized waveforms results in a peak bipolar MUAP amplitude of 0.87904 units, which is depicted immediately below to reinforce that it is the result of a sample-by-sample summation of G1 plus G2 (blue circle).

The distance between G1 and G2 in panel A is large enough that the depolarization recorded at G1 is summed with the smaller amplitude of the leading edge recorded at G2. The resulting bipolar MUAPs resemble two separate monophasic waveforms that are opposite in polarity. Notice that the non-propagating components recorded at G1 and G2 are similar in shape but opposite in polarity. As G1 is moved toward G2, the amplitude of the non-propagating component recorded at G1 increases, resulting in a progressively diminished bipolar EOF effect when the two signals are summed together (panels A–D). Thus, while greatly reduced, this example illustrates that the high-frequency crosstalk associated with the non-propagating component cannot be eliminated. High-frequency crosstalk is even present in a double differential (DD) signal where a tripolar electrode (i.e., G1, G2, and G3) allows for two sets of bipolar recordings that can be subtracted from each other to obtain a second difference [46].

### 5.2. Laying the Foundations for Spatial Filtering

Figure 7 shows that the MUAP recorded at G1 developed a leading edge as the electrode is placed closer to G2. The leading-edge increases in length as the emergent tripole must propagate farther to reach G1 (panels B–D, top). There is a concomitant increase in the overlap between the leading edges recorded at both G1 and G2 with decreases in the distance between the two electrodes. Summation then results in a progressively flatter baseline for the bipolar MUAP (panels B–D, bottom). When G1 is close enough to G2, the depolarization phases start to overlap and begin to cancel, making the bipolar MUAP appear purely biphasic (panel D). Partial cancellation occurs because portions of the same phase span both G1 and G2, which also reduces the peak amplitude of the bipolar MUAP. Thus, as the distance between G1 and G2 decreases, the MUAP recorded at G1 becomes spatiotemporally more similar to the MUAP recorded at G2. Theoretically, the MUAP recorded at G1 and G2 would be identical in time and space if they could occupy the same location (panel E). The summation would result in cancellation. In practice, the cancellation effects “do” occur when G1 and G2 are placed close together with the IZ in between them (“straddling” the IZ). Since MUAPs propagate bidirectionally away from the IZ, G1 and G2 record mirror images of the same MUAP, which results in cancellation effects [60,67,78,79]. A bipolar electrode configuration has G1 and G2 placed side-by-side with an IED between 1 and 2 mm, in line with the muscle fibers between the IZ and tendon. The first electrode should be at least 10 mm away from the IZ [7]. 

Before the point at which cancellation effects begin in Figure 7, there is a critical distance between the two electrodes at which the peak of the repolarization phase aligns with the location of G1, and the peak of the depolarization phase aligns with the location of G2 (panel C, top). The distance between MUAP peaks is the same as the distance between G1 and G2, resulting in a bipolar MUAP that has the greatest peak amplitude (panel C, bottom). As will be detailed further, Basmajian and DeLuca [80] showed that when the distance between waveform peaks is the same as the distance between electrodes, the bipolar signal will be the result of the “perfect addition”. The distance between G1 and G2 that results in perfect addition is illustrated in Figure 7C, and it is foundational for understanding other aspects of EMG theory.

### 5.3. Qualitative Frequency Analysis of Bipolar MUAPs

Digital or analogue filtering of a signal changes both its amplitude and frequency characteristics. Similarly, a bipolar electrode configuration is considered as a “spatial” filter because the amplitude and frequency characteristics of the surface EMG signal depend on the spatial distance between G1 and G2 [8]. Changes in the amplitude and frequency of the bipolar MUAP obtained by the summation of monopolar signals recorded at G1 and G2 at progressively smaller distances between them illustrate spatial filtering in an obvious way.

Recall that the frequency of a sinusoid is based on the number of cycles (upward and downward phases) that are completed in one second; a higher frequency sinusoid has more cycles within one second than a lower frequency sinusoid. Now consider the bipolar MUAP on its time scale (20 ms), where the time between the downward and upward phases is the duration without the non-propagating component. As the distance between G1 and G2 decreases, the duration of the bipolar MUAP is also decreasing. Using the definition of the frequency of a sinusoid, the bipolar MUAP becomes a higher frequency waveform because more cycles “could” fit within the 20 ms period. At the same time, the *V_PP_* of the bipolar MUAP exhibits increases and decreases depending on the distance between G1 and G2. The surface EMG IP exhibits the same spatial frequency effects due to the distance between electrodes, but the amplitude and frequency changes are easier to illustrate using the bipolar MUAP. Taken together, Figure 6 and Figure 7 should reinforce why it is critical to control for electrode location and the distance between electrodes across test sessions to ensure that changes in the surface EMG signal are *not* due to variations in these two critical factors.

## 6. Common Mode Interference

An interesting question raised by students is: If placing G1 and G2 close to each other on the muscle alters the amplitude and frequency content of the surface EMG signal, what is the benefit of a bipolar electrode configuration? The answer involves an understanding of the nature of power line interference (PLI) at 50–60 Hz. Kinesiological surface EMG is usually not performed in an electrically isolated environment, as would occur in a room designed for electrodiagnostic testing. As a result, the electromagnetic field created by the PLI is detected by G1 and G2 at the same time, which now has a 50 Hz or 60 Hz signal that both electrodes share in common (“common mode”). A voltage gradient is then created so that there is a potential difference between any two points on the body. Although the PLI voltage frequency of 50 Hz or 60 Hz will be present in both electrodes at the same time, a smaller IED (<10 mm) ensures that the potential at both G1 and G2 is similar, so that the unwanted differential source is reduced [81,82]. The distance between G1 and G2 that occurs with a monopolar configuration can be quite significant, increasing the possibility that the voltage gradient at G1 is different from that at G2. 

**Figure 8:**
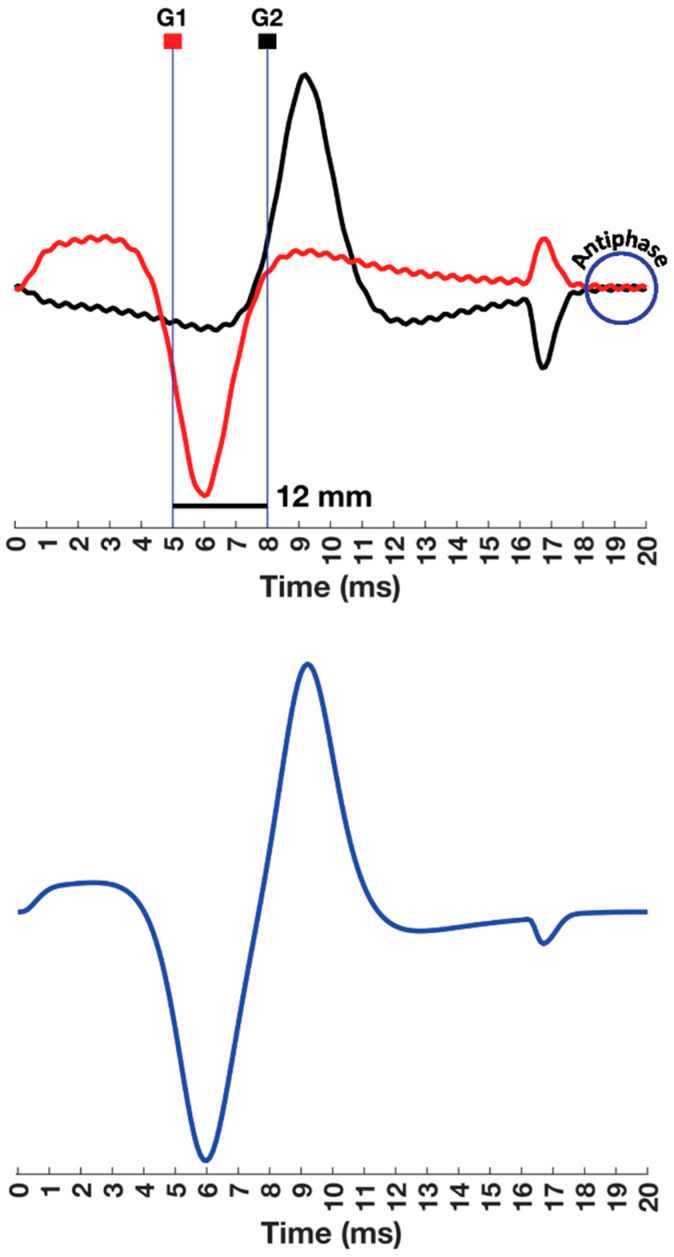
An illustration of common mode rejection associated with the bipolar electrode configuration. Continuing with the approach depicted in Figure 7, two monopolar compound muscle action potentials (CMAPs) are presented in the top panel. The CMAPs recorded by G1 (red) and G2 (black) are contaminated by a common mode signal. The signal recorded by G2 is inverted, so it is antiphase to that present in G1 and is cancelled (bottom panel). Since the time scale of the MUAP is 20 ms, a small amplitude 2 kHz sinusoid was created to illustrate a common mode signal that could also be observed in the baselines of G1 and G2 after the muscle fiber–tendon end effects.

The difference in PLI cancellation between monopolar and bipolar electrode configurations is illustrated in Figure 8, which is an idealized representation. Unfortunately, electronic circuitry results in a less than perfect cancellation that is reflected in the common mode rejection ratio (CMMR) of the amplifier. The CMMR of an amplifier is always listed in the equipment specifications and it reflects how well the amplifier reduces the *V_PP_* of the signal that is common to both electrodes. A CMMR of 10,000 means that the ratio between the differential voltage and the common mode voltage at the output of the amplifier has been reduced by 10,000 times. For example, if the common mode voltage and the differential voltage are equal at the input (ratio = 1) but the common mode voltage is amplified 0.01 times while the differential voltage is amplified 100 times, the ratio of the output will be 10,000. The CMMR is the differential gain divided by the common mode gain, and it is usually expressed in decibels to make a direct connection with signal energy: 20 log_10_ (10,000) = 80 dB. The acceptable standard for a surface EMG amplifier is 100 dB [83].

Wire loops such as electrode leads are suspectable to electromagnetic field-induced current, which is often overlooked. The larger the loop area, the larger the current induced by the power line magnetic field. For this reason, wires are twisted to reduce the area [83,84]. Twisting the leads of G1 and G2 in a close bipolar configuration is much easier to accomplish than in a monopolar configuration, which is another factor that makes the monopolar electrode configuration more susceptible to PLI [84].

The efficacy of the amplifier to subtract-out the common mode signal depends on G1 and G2 recording the same amplitude at the same time. Thus, the inputs from the leads into the amplifier must be balanced. Balancing the inputs into the amplifier involves matching the skin-electrode input impedance at G1 and G2. Careful cleaning of the skin surface and judicious use of an electrolyte gel can ensure that the skin-electrode input impedance for G1 and G2 are equally low [83,85]. Amplifiers are tolerant to some degree of imbalance, but the skin-electrode input impedance should be measured as a methodological control [86,87]. This ensures that “both” the amplitude and phase of PLI recorded by G1 and G2 are the same [88]. Matching the skin-electrode impedance is more easily accomplished when both G1 and G2 are close together in a bipolar configuration [88].

## 7. Volume Conduction

While the quadrupole is a more accurate representation, a current tripole representation of the MFAP detected by a point electrode is used to motivate the relationship between IED and the wavelength (λ) in the same way that a monopolar electrode configuration was used to lay the foundation for spatial filtering. Furthermore, the detection of a dipole by a point-electrode is familiar to undergraduate students and the geometry lends itself well to analogies that can be used for an intuitive understanding of more complex ideas outlined in the following sections. 

### 7.1. Visual Field and Electrode Pickup Volume

**Figure 9:**
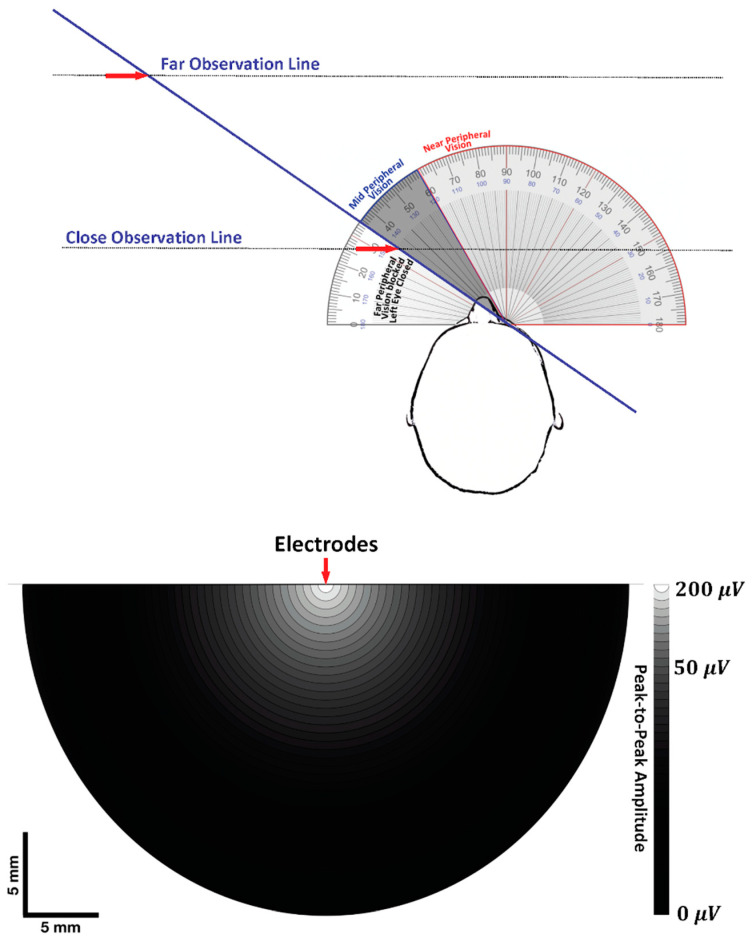
A comparison between the visual field (top panel) and electrode pickup volume (bottom panel). The top panel shows the monocular visual field for the right eye, with the left eye shut. The horizontal black dotted lines indicate the geometry of object detection for near- versus far-visual fields of observation as an object moves from left-to-right mid- to near-peripheral vision and then center gaze. The red arrows denote the point at which the object is first detected at both observation distances. The bottom panel shows the pickup volume of bipolar electrodes with an interelectrode distance (IED) of 6 mm. The voltage gradient is mapped onto a cross-section of the muscle, normal to the fibers. The G1 electrode is behind G2, in parallel with the muscle fibers, at the origin of the voltage gradient. The voltage gradient is based on the peak-to-peak amplitude (*V_PP_*) of the motor unit action potentials (MUAPs) detected by surface electrodes that correspond to the depth at which that same MUAP was detected by a needle electrode. The greatest *V_PP_* is white on the greyscale voltage gradient, with darker shades indicating a progressive decrease with increasing distance away from the surface electrodes.

The following paragraphs lay the foundation of an analogy that is made between the visual field and the pickup volume of surface electrodes (Figure 9). The top panel of Figure 9 shows the monocular visual field for the right eye, with the left eye shut [89]. The analogy is designed to make a connection that the magnitude and duration of the visual stimulus depend on where the target is located within the visual field. Figure 9 also shows the geometry of object detection for near- versus far-visual fields of visual observation as it moves from left-to-right, mid-to-near-peripheral vision, and then center gaze [90]. The red arrows denote the point at which the object is first detected for both observation distances.

The bottom panel of Figure 9 shows the pickup volume (visual field) for bipolar electrodes based on the work of Lynn and Beatty [91], where the voltage gradient is mapped onto a cross-section of the muscle, normal to the fibers. The electrodes are at the center, with G1 behind G2 in parallel with the muscle fibers. The values were taken from the work of Roeleveld et al. [92], where the *V_PP_* of the surface detected MUAPs were measured in relation to those same MUAPs identified by a needle electrode at specific depths within the muscle. The surface electrodes had an IED distance of 6 mm while the authors inserted a needle electrode into the muscle at different depths up to 30 mm.

The radius of the hemisphere presented in Figure 9 is limited to 20 mm because the heat map is completely black at this distance because surface MUAPs cannot be detected from MUs at that depth and beyond. It is known that the signal strength generally exhibits a rapid non-linear decrease with an increased distance away from the electrode [93,94,95,96]. The heat map shows that the *V_PP_* of the surface MUAPs decreases from 200 μV to 50 μV at an approximate depth of 6 mm, with a dramatic asymptote after that depth (see Figure 9). These findings clearly show that the radius of the hemisphere that defines the pick-up volume of tissue is related to the center-to-center distance between the electrode detection surfaces (IED = 6 mm in this case). The radius of the pick-up volume is also determined by the *V_PP_* electro-skin junction noise (on the order of 5–20 μV) and by the *V_PP_* referred-to-input (RTI) noise of the amplifier (typically 3–4 μV), above which MUAPs can be meaningfully detected [94,97]. Note that, if modern amplifiers are used, the most important noise level limiting the detection of MUAPs is the electrode-skin noise and not the amplifier noise.

### 7.2. Trying to Cross the Street with One Eye Shut

**Figure 10:**
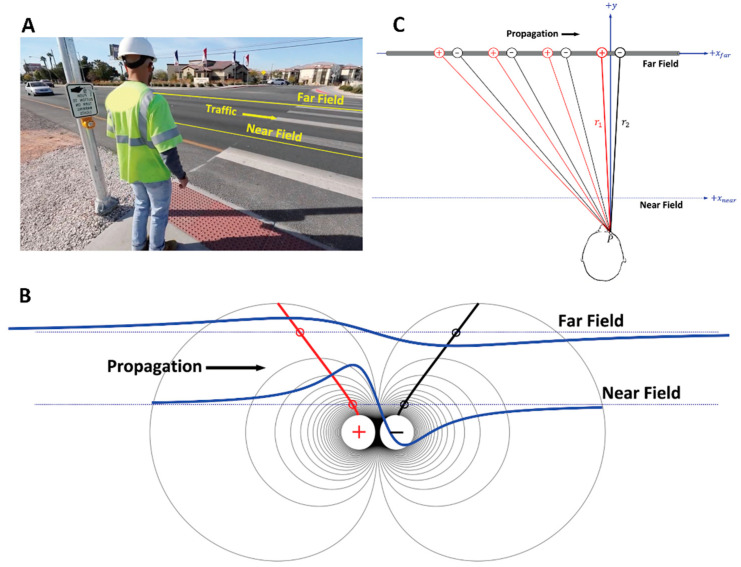
An illustration of the parallel between how the size and shape of an object depend on where it is located (near versus far) relative to the observer, that is, the electrode detection surface. An individual is waiting on the edge of a curb to cross a three-lane highway (panel (**A**)), equipotential lines associated with a dipole (panel (**B**)), and the geometry of a propagating dipole detected by a point-electrode (panel (**C**)) are linked together in the analogy. In each panel, horizontal dotted lines indicate near and far observation distances of the target moving from left to right, which is a delivery van travelling in panel A and the muscle fiber action potential (MFAP) in the panels (**B**,**C**).

Figure 10 sets the stage for the analogy. Imagine standing on the edge of a curb, waiting to cross a multilane highway (panel A). Equipotential lines associated with a dipole (panel B) and the geometry of a propagating dipole detected by a point-electrode (panel C) are linked together in this analogy. In each panel, horizontal dotted lines indicate near and far observation distances of the target moving from left to right, which is a delivery van travelling in panel A and the MFAP in panels B and C. The time interval during which the van is in front of the viewer is directly proportional to its length and inversely proportional to its speed, and the same holds for a propagating tripole.

#### 7.2.1. Case 1: Near-Field Observation

The analogy is that of stepping off a curb to cross the road, becoming startled, then suddenly having to pull back due to inattention, and not looking right or left before starting to cross the road. To recreate that experience, close the eye on the side of the body that would be the first to see approaching cars (left in North America). The head and neck are looking down at the road, a few strides in front of the feet. Head movement is restricted to only looking forward. The first case is a small delivery van approaching in the closest lane, only 30 cm away from the curb. The van seems to appear out of nowhere, becoming like a huge wall in front of the pedestrian, then disappears just as quickly as it suddenly appeared. The velocity of the van is such that the observer is “just” able to catch sight of the front wheel (depolarization) and then the back wheel (repolarization). A plot of the visual stimulus intensity for monocular perception (monopolar electrode) of the van as a function of time would resemble the near field MFAP (see panel B).

#### 7.2.2. Case 2: Far-Field Observation

The second case is the delivery van approaching in the third lane (≈15 m). As a result, the van is perceived much sooner (panel A). Even though it is going at the same speed, more distant objects appear to travel more slowly. There is a longer time lapse between seeing the front (depolarization) and back (repolarization) wheels. Moreover, the van no longer seems like a wall when it reaches the central gaze. At a farther distance, the entire dimensions of the van can be observed. Thus, the van also appears smaller than before. A plot of the visual stimulus intensity of the monocular perception (monopolar electrode) of the van as a function of time would look like the far field MFAP depicted in panel B. Notice that the second MFAP is lower in amplitude (visual stimulus) and longer in duration (perceived earlier from farther away).

#### 7.2.3. The Geometry of Electrode-Source Detection

At a certain distance away, the entire delivery van fills the field of vision perfectly. Both wheels are equidistant from the center line of vision. Since the front and back wheels represent the depolarization and repolarization phases in this analogy, hypothetically, both poles cancel and there would be a momentary “blind spot” (zero-crossing) dead center between them. The geometry of how a point-electrode detects a propagating dipole can now be considered [70,96,98]. The length of the lines connecting the dipole to the electrode as it propagates along the muscle fiber is presented in Figure 10, panel C. While the MFAP is propagating from left to right, the negative charge is always closer to the electrode (r_2_ < r_1_). The potential is progressively becoming more negative, reaching a peak when directly underneath the detection surface, then decreasing toward zero. The zero crossing occurs when both charges are equidistant from the observation point-electrode and the muscle fiber (figurative blind spot). As the MFAP continues to propagate toward the right, the trailing positive repolarization phase is now closer to the electrode (r_1_ < r_2_) and the potential is becoming progressively more positive, reaching a peak, then declining toward zero as its distance on the right side of the electrode increases.

#### 7.2.4. Dipole Field Lines and Apparent Distance between MFAP Peaks

The field lines associated with a dipole can now be connected with changes in the amplitude and duration of the MFAP due to electrode-source distance (panel B). Each point along a line denotes the electric potential at a point (x,y) in extracellular space. There is a nonlinear increase in the distance between each successive radial line extending from the dipole center. At the same time, there is a nonlinear decrease in electric potential with each successive interval as the current flows through the extracellular fluid to the skin surface. The decrease in *V_PP_* is quite rapid as depicted in the bottom panel of Figure 9. The red and black lines connect the maximum (x,y) points for each successive radial line for the positive (repolarization) and negative (depolarization) charges, respectively.

The two horizontal lines in panel B correspond to the near and far electrode-source distances. As the dipole propagates from left to right, the intersection of the red and black lines with the field lines indicates where the peak positive and negative values are located at each observation distance. It is critical to notice that the distance between the positive and negative peaks is increasing, with increasing radial distance away from the two charges. It appears that the physical distance between charges on the muscle fiber is increasing, but it is the result of the filtering due to the volume conduction effect. It is important to make the connection between the red and black lines showing the distance between the positive and negative peaks and electrode-source distance effects on MFAP amplitude and duration. These effects form the basis for understanding the more complex theory described in the literature [99].

#### 7.2.5. Distance between MFAP Peaks, Interelectrode Distance, and Selectivity

The same principles of volume conduction apply to bipolar detection, but certain aspects of the geometry have changed because there are two detection surfaces side-by-side. There is a critical electrode-source distance at which volume conduction has increased the apparent distance between the positive and negative MFAP peaks so that it is equal to the IED. When the dipole propagates underneath both electrodes, the positive charge is directly under G1 and the negative charge is directly under G2 at the same time, so there is perfect addition, as depicted in Figure 7C. Because the apparent distance between the poles is a function of how far away the muscle fiber is from the electrode, perfect addition continues as the MFAP propagates toward the distal ends. Increases in the electrode-source distance expand the distance between the positive and negative MFAP peaks, increasing the degree of overlap of the same phase across G1 and G2 as it propagates along the fiber. The degree of cancellation increases. These effects are analogous to what was described for decreases in the distance between G1 and G2 while the electrode-source distance remained constant (Figure 7).

In this way, a small IED can be used to selectively record muscle fibers that are closer to the electrodes where a dipole distance closely matches the IED. An IED of up to 10–20 mm is recommended for larger or deeper muscle groups (i.e., biceps). In the case of smaller muscles, the IED should not exceed one-quarter of the muscle fiber length [7] or 10 mm, whichever is smaller [8]. Data are available on the muscle fiber lengths for both the large and smaller muscle groups (e.g., [100,101,102]) so it does not have to be estimated. Smaller IEDs (≤5 mm) are used for MU identification [103,104]. The electrodes will selectively record the closest muscle fibers. The number of MUs contributing to the signal is therefore limited, thereby reducing the complexity of the surface EMG IP by attenuating lower amplitude volume conducted potentials from farther away. A less complex IP allows for easier identification of the different MUAP shapes from fibers within the “pickup volume” [103,104]. The latest innovations involve high-density surface electromyographic (HD-surface EMG) grid electrodes (IEDs <5 mm) that record the propagation of MUAPs across a large surface area over the muscle to allow for new insights into the control and regulation of muscle force [105].

## 8. Wavelength

Muscle fiber conduction velocity determines the time duration that the MFAP is detected by the electrode. The propagation of an MFAP with a higher MFCV will be detected by the electrode for a shorter period, which decreases its duration. Since MFAP duration is determined by its MFCV, the degree of overlap of the same phase across G1 and G2 is also determined by MFCV [106,107,108]. The effects of MFCV on addition versus the attenuation of MFAPs for any given IED is based on the wavelength (λ) of the signal [80]. This topic has traditionally been presented using the mathematical relationship between the frequency of a sinusoid propagating in space (like a sea wave) and its wavelength (λ), which is the spatial extent at which a specific point on the periodic waveform starts to repeat [8,109]. The demarcation could be any point, but the crests (or, peaks) are chosen because they are easily identifiable. While it is an eloquent approach, the goal of this review is to provide a concrete visual example before providing the mathematical formalism.

## 9. Conduction Velocity and Wavelength

**Figure 11:**
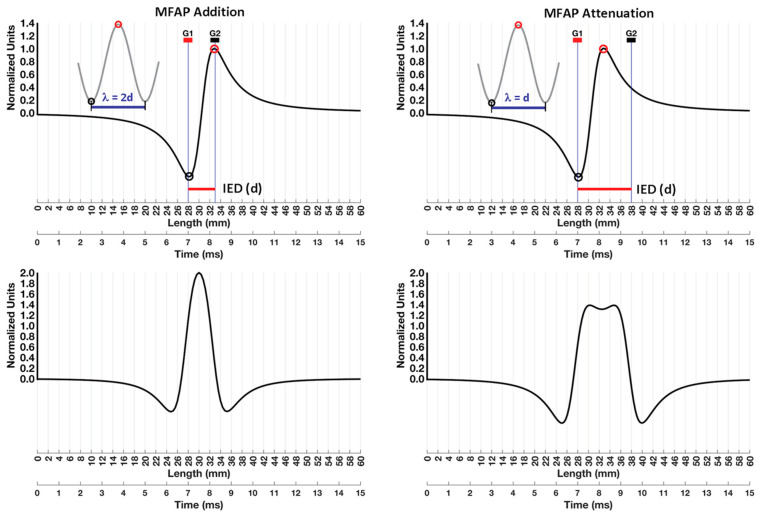
An illustration of the interaction of wavelength (λ) and interelectrode distance (IED). The top left panel shows the muscle fiber action potential MFAP relative to G1 and G2 with an IED of 5 mm. The depolarization and repolarization peaks align with G1 and G2, respectively. The amplitude is normalized so that the maximum positive value is 1 unit. The grey sinusoid depicts the perfect addition wavelength (λ). The resulting bipolar MFAP has a peak amplitude of two units (bottom left panel). The top right panel shows the same MFAP but now the IED has been increased to 10 mm. The grey sinusoid depicts the wavelength (λ) associated with attenuation. Under these conditions, the bipolar MFAP experiences attenuation (bottom right panel). The peak amplitude of the bipolar MFAP was reduced to 1.438 units.

Figure 11 illustrates the interaction of wavelength (λ) and interelectrode distance (IED). The muscle fiber action potential (MFAP) that is depicted is due to a symmetric dipole. The non-propagating muscle fiber–tendon end effects have been omitted to create a simple visual depiction of the addition and attenuation of wavelengths (λ’s). For each graph, the first *x*-axis corresponds to space (mm) between the innervation zone (x = 0 mm) and the muscle-tendon junction (x = 60 mm). The second *x*-axis is the time-evolution of the MFAP in milliseconds for a conduction velocity of 4 m/s. The use of two scales only applies to travelling potentials generated by moving sources. 

### 9.1. Addition

Figure 11 (top left) shows an MFAP that is represented by a simple dipole with symmetric depolarization and repolarization phases to facilitate the connection with the definition of wavelength (λ). The IED was 5 mm. For a muscle fiber that is close to the electrode, an MFCV of 4 m/s results in a 5 mm distance between the positive and negative MFAP peaks. As a result, when the peak of the depolarization phase has reached G2, the peak of the repolarization phase has reached G1. The result is a perfect addition (bottom left). There was a two-fold increase in the peak amplitude of the bipolar MFAP. The situation was the same as that presented in Figure 7C.

The grey sinusoid in the top left panel illustrates the definition of wavelength (λ) associated with perfect addition [80]. Note the correspondence between the black and red circles on the peaks on the wavelength (λ) and the depolarization and repolarization phases of the MFAP. The grey dotted line (traditionally not represented as a dotted line) signifies the extension of the curve to complete the wavelength (λ) definition, which is the point where a periodic waveform begins to repeat itself. The distance between peaks of the depolarization and repolarization phases was equal to the IED (or d). The distance between the first and second bottom peaks of the wavelength corresponded to twice the IED (λ = 2d). Thus, the depolarization and repolarization peaks aligned with G2 and G1 (respectively) for perfect addition when the wavelength (λ) equaled 2d.

### 9.2. Attenuation

The right panels of Figure 11 show an IED (or, d) for which the MFAP attenuation occurred. The MFCV was 4 m/s, but now the IED increased to 10 mm. Note that the distance between peaks of the depolarization and repolarization phases (which was still 5 mm) was now half the length of the IED (or d). The wavelength (λ) that the MFAP “could” be considered to start repeating is now equal to the IED (or d). The grey sinusoid presented in the top right panel was designed to reinforce the spatial relationship between the MFAP peaks, the IED, and the attenuation wavelength (λ = d). The amplitude of the bipolar MFAP is depicted immediately below; this was attenuated compared to the bipolar MFAP in the bottom left panel.

Recall that Section 5.3 showed that a decrease in distance between G1 and G2 resulted in a decrease in the amplitude and an increase in the frequency of the bipolar MUAP (see Figure 7C). In that example, the duration of the bipolar MUAP was linked to its frequency. Thus, there is another way to express the changes associated with the IED that also applies here: periodic waveforms with the same frequency as the bipolar MFAP will also be attenuated at this particular IED. Conversely, if the bipolar MFAP has experienced addition, periodic waveforms with that same frequency will also be amplified (Figure 11, left panels). A more extended reading on how IED affects the amplitude and frequency content of the bipolar surface EMG signal may be found in Merletti and Muceli [8], who used harmonic analysis along with more sophisticated visual descriptions. 

It is important to bear in mind that this example of attenuation was simplified in another way. Electrode-source distance and MFCV were held constant while only the IED was changed. In reality, the IED is held constant while the electrodes detect muscle fibers at different depths within the pickup volume, where the normal distribution of MFCVs ranges between 3 and 6 m/s [30,31,110]. Thus, for any given IED, some MFAPs within the pickup volume will experience addition while others will be attenuated. The mathematical relationship between MFCV, IED, and wavelength (λ) that predicts the outcomes of spatial filtering should now be more accessible due to the creation of concrete images of addition and attenuation. 

## 10. Factors Affecting the Analysis and Interpretation of the Surface EMG Signal

### 10.1. Global Surface EMG Measures

The interpretation of the surface EMG signal by kinesiologists and physical therapists is often focused on functional assessment and/or alterations associated with an intervention. The onset and magnitude of the surface EMG signal are used as global measures of coordination within and between muscle groups and the force generated by the muscle, respectively [111]. Those who are new to surface EMG must always be aware that the signal is affected by both the technical factors associated with the electrode detection system (i.e., IED and impedances) and the peripheral factors associated with the muscle that influence MFCV (i.e., temperature and fatigue). As a result, interpretation of the surface EMG findings must be made knowing that factors that affect the surface EMG signals that do not reflect alterations in neural drive to the muscle [86,87,112,113,114,115,116].

Previous sections illustrated that the electrode-source distance affects the amplitude and frequency content of the surface EMG signal, which also applies to the impact of subcutaneous tissue. There is a strong negative correlation between the skinfold thickness and surface EMG amplitude and mean power frequency [117]. In addition, increased MU recruitment and discharge frequency associated with the gradation of muscle force results in a progressive increase in the cancellation effects between MUAPs within the surface EMG IP. As a result, the relationship between the surface EMG amplitude, neural drive to the muscle, and force output is more complicated than previously thought [116,118,119]. There is also increasing evidence that training-related changes in muscle size can alter the surface EMG signal [120]. Consensus papers by ISEK address the various techniques that can be used to facilitate interpretation of the surface EMG signal against the backdrop of the complicating factors outlined above [11]. There are also statistical considerations involved in methods used to compare the surface EMG signal long-term interventions and between populations [47,121,122,123].

### 10.2. Surface Detected Motor Unit Activity

High-density electrode grids (HD-surface EMG) now allow for the direct monitoring of MU recruitment and discharge rates non-invasively from the skin surface. This major advance now allows for the interpretation of changes in the neural control of muscle that eludes the more global measures obtained from bipolar detection [124,125]. Quantification of surface MUAPs offers greater specificity, which is required for monitoring neuropathic and myopathic disorders [126,127,128,129]. However, it is important to bear in mind that the same peripheral factors that would affect the interpretation of global measures of surface EMG magnitude would also affect the *V_PP_* of the detected MUAPs. The same methodological controls apply for correct interpretation.

Kinesiologists and physical therapists are taught Henneman’s size principle in relation to MU size and its biochemical characteristics (Type 1, IIA, and IIB) [130]. Since there is no way to verify the muscle fiber type using the surface EMG signal, students must transition to the concept that MUs are identified based on a specific percentage of maximal voluntary contraction (MVC) at the time it is recruited. For example, a low threshold MU is recruited at a lower percentage of MVC versus a high threshold MU that is recruited at a higher percentage of MVC; they would not be referred to as slow- and fast-twitch fibers, respectively [131]. Innovative research using HD-surface EMG is now documenting differential adaptations for low- versus high-threshold MUs in response to diet and resistive exercise [132] and pain exposure [133]. In practice, the sample MUs are therefore delineated based on the interval over which they were recruited. When a graded categorization has been required, investigators have evaluated changes in MU activity over discrete 20% MVC intervals [132]. However, a broader definition has also been used where lower threshold MUs are those recruited between 0 and 35% MVC and higher threshold MUs are those recruited between 35% to 70%, or higher [134].

## 11. Summary and Conclusions

A combination of analogies, visual descriptions, and qualitative analysis of biophysical concepts was presented to develop an intuitive understanding of foundational concepts in surface EMG theory. Differences in height between two individuals were used to introduce differential amplification. Internal and extracellular recordings illustrate that potential differences between two points were made with respect to a common reference. Extracellular recordings were then extended to monopolar surface recordings of the muscle fiber action potentials (MFAPs), where a connection was also made with the spatiotemporal representations of action potentials. The approach involves understanding the potential that is recorded by an electrode as it is moved to different locations along the skin surface above the muscle with tripole field lines between the muscle and skin surface. The current tripole was extended to the quadrupole to introduce the muscle fiber–tendon end-effects and explain the non-propagating component of the MFAP. Changes in the propagating and non-propagating components of the motor unit action potentials (MUAPs) are detailed as a monopolar electrode was moved between the innervation zone (IZ) and tendon and as a function of the muscle fiber depth. Bipolar recordings were introduced as two separate, time-delayed monopolar recordings of MUAPs that were subtracted from each other. Changes in the shape of the bipolar MUAP are described as the distance between the two monopolar electrodes, which were progressively decreased to provide a foundation for spatial filtering. The critical distance between the monopolar electrodes that resulted in the greatest peak-to-peak amplitude (*V_PP_*) for the bipolar MUAP was highlighted. The distance where the *V_PP_* for the bipolar MUAP started decreasing was also identified.

Spatial filtering was discussed in relation to common mode signals, with considerations for both monopolar and bipolar recordings. An in-depth examination of volume conduction was provided through an analogy between the detection of targets with visual field and electrode pick-up volume. The geometry of electrode-source detection at near and far distances was connected with dipole field lines. The analogy also showed how the dipole field lines explain the spatial expansion of positive and negative MFAP peaks, as the action currents flow away from the dipole toward the electrode. A detailed visual description of wavelength (λ) was used to describe what frequencies are passed or attenuated by a particular IED. Because of the ease of use of surface EMG techniques, it is important to introduce students to factors that affect the analysis and interpretation of the surface EMG signal early in the curriculum to prevent the establishment of inappropriate practices [76].

This work introduced fundamental concepts through analogies that could be illustrated by teachers to students of physiotherapy and kinesiology having heterogeneous backgrounds and a lack of familiarity with physical and mathematical approaches. Together with the work of Campanini et al. [109] in this Special Issue, the present paper is expected to provide teaching tools that would facilitate the understanding of more complex published tutorials, guidelines, best practices, and consensus papers, and make them approachable by students and clinical operators in the field.

## Data Availability

Not applicable.

## Abbreviations

EMG	Electromyogram/electromyography
sEMG	Surface electromyogram
HD-sEMG	High-density sEMG
IZ	Innervation zone of a motor unit or muscle
IP	Interference pattern
MU	Motor unit
MUAP	Motor unit action potential
NMJ	Neuromuscular junction
IED	Interelectrode distance
MFAP	Muscle fiber action potential
CMAP	Compound muscle action potential
M-wave	Motor wave
MP	Motor point
G1	Grid Electrode 1 (E1)
G2	Grid Electrode 2 (E2)
GND	Ground
EMF	Electromagnetic fields
ISEK	International Society of Electrophysiology and Kinesiology
CV	Conduction velocity
MFCV	Muscle fiber conduction velocity
NCV	Nerve conduction velocity
LD	Leading dipole
TD	Trailing dipole
SD	Single differential
DD	Double differential
*V_PP_*	Peak-to-Peak Voltage Amplitude
PLI	Power line interference
CMMR	Common mode rejection ratio
